# Hand Positions Alter Bistable Visual Motion Perception

**DOI:** 10.1177/2041669516651379

**Published:** 2016-06-17

**Authors:** Godai Saito, Jiro Gyoba

**Affiliations:** Tohoku University, Sendai, Japan

**Keywords:** stream/bounce perception, proprioception, embodied perception

## Abstract

We found that a hand posture with the palms together located just below the stream/bounce display could increase the proportion of bouncing perception. This effect, called the hands-induced bounce (HIB) effect, did not occur in the hands-cross condition or in the one-hand condition. By using rubber hands or covering the participants’ hands with a cloth, we demonstrated that the visual information of the hand shapes was not a critical factor in producing the HIB effect, whereas proprioceptive information seemed to be important. We also found that the HIB effect did not occur when the participants’ hands were far from the coincidence point, suggesting that the HIB effect might be produced within a limited spatial area around the hands.

Here, we report the effect of the observer’s hand postures on the interpretation of a bistable visual motion event. In the stream/bounce display, two identical moving objects approach, coincide, and then separate producing the ambiguous and bistable perception that the two objects either stream through or bounce off each other (Michotte, 1946/1963). Observers have been reported to normally perceive streaming more frequently than bouncing (Bertenthal, Banton, & Bradbury, 1993). However, research on multisensory interaction has indicated that external signals, such as additional visual and auditory stimuli, alter the bistable motion perception (Kawachi & Gyoba, 2006; Sekuler, Sekuler, & Lau, 1997; Watanabe & Shimojo, 2001). Expanding on these findings, we examined whether specific hand postures, such as the palm-to-palm posture, would facilitate bouncing perception, since previous studies have indicated that hand posture could modify visual perception in several ambiguous situations (e.g., Wohlschläger, 2000).

The participants’ task was to watch the stream/bounce display from a distance of 40 cm with their head on a chin rest (Figure 1a), and to report verbally whether the two moving objects appeared to stream through or bounce off each other. Six pictures (c1, e1, e2, e3, e4, and c2) in Figure 1(b) show the six conditions used in Experiment 1. In the two control conditions, occurring first (c1) and last (c2), the participants rested their hands on their lap (no hands condition). There were four experimental conditions. In the hand-leftward condition (e1), the participants put the palm of their right hand on the monitor and rested their left hand on their lap. In the hands-together condition (e2), the participants put their palms together. In the hands-leftward condition (e3), the participants put the back of their left hand and the palm of their right hand together. In the hands-cross condition (e4), the participants crossed their hands by putting the back of their hands together. The four conditions were applied in a random order for the participants. Following a practice session of 10 trials using the no hands condition, all participants were presented with 300 trials (6 conditions × 50 trials). The results (Figure 1b) indicated that only the posture with one’s palms together promoted the bouncing perception. Hereafter, this phenomenon is referred to as the hands-induced bounce (HIB) effect. Subsequently, in Experiments 2, 3, and 4, we attempted to explore possible critical factors responsible for producing the HIB effect.

**Figure 1.**
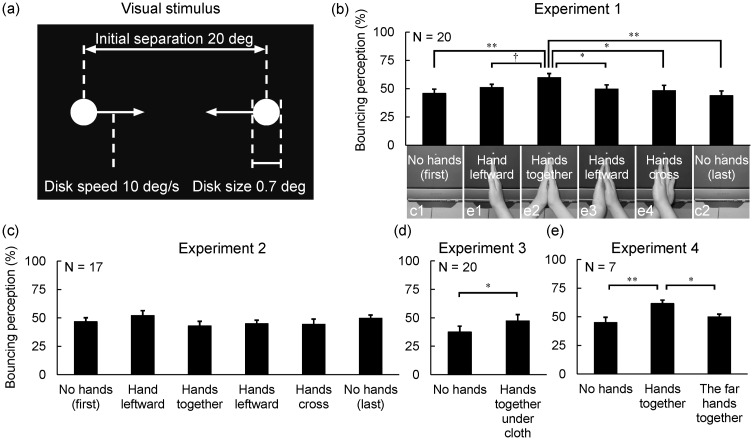
(a) Stimulus configuration in experiments. (b to e) Mean percentages of bouncing perception in each condition of Experiments 1 to 4. Percentages were compared using a one-way repeated measures analysis of variance. Experiment 1: *F*(5, 95) = 2.44, *p* < .05, $n_{p}^{2}$^ ^= 0.11; Experiment 2: *F*(5, 80) = 1.26, *p* = .30, $n_{p}^{2}$^ ^= 0.07; Experiment 3: *F*(1, 19) = 5.66, *p* < .05, $n_{p}^{2}$^ ^= 0.23; Experiment 4: *F*(2, 12) = 7.30, *p* < .01, $n_{p}^{2}$^ ^= 0.55. We used Ryan’s method for multiple comparisons due to its universal applicability (Horn, 1981). Error bars represent the standard error of the mean. ^†^*p* = .08, *p < .05, **p < .01.

In Experiment 2, a set of rubber hands, instead of the participants’ own hands, was placed in the same manner as in Experiment 1. The participants were asked to keep their arms folded during the observation, so that they received visual information from the postures of the rubber hand(s), while the proprioceptive and tactile information from their own hands was held constant. This manipulation was also intended to prevent the perception of hand ownership. The results (Figure 1c) showed that the sight of a rubber hand did not influence the perception of visual stimuli.

In Experiment 3, the participants in the hands-together condition put their palms together underneath a black cloth just below the coincidence point of the stream/bounce display. The no-hands condition (hands resting on lap) was used as the control condition. The results (Figure 1d) showed that the HIB effect could also occur when the proprioceptive and tactile information from the participants’ palms being together was present, even without visual feedback from their own hands.

In Experiment 4, the no hands, the hands-together, and the far hands-together conditions were used. In the far hands-together condition, the participants put their palms together at approximately 21° below the coincidence point of the stream/bounce display. The results (Figure 1e) revealed that the HIB effect only occurred when the participants’ hands were just below the coincidence point, but did not occur when their hands were far from this point, despite the tactile information derived from the palms being the same in both conditions.

There are several possible interpretations of our results. First, there is a likelihood that the posture with the palms together could produce a sort of top–down bias increasing bouncing perception. However, the top–down bias is generally assumed to occur at a later stage of processing, after the modality-specific sensory processing. As our results have modality-specific and spatially limited properties, this suggests that response bias plays little role in the HIB effect and could not alone account for all our results.

A second alternative is the possible influence of additional nearby visual objects. For example, in the no-hand condition of Experiment 3, the black cloth was absent, and therefore, the visual display was different. This is also the case with the far condition of Experiment 4. Indeed, the bouncing percept in Experiment 2 was generally more frequent than in Experiment 3. Yet, the effect of additional nearby visual objects cannot explain the results of Experiment 1, where the hands-together condition showed a significantly higher rate of bouncing perception compared with the hands-leftward and the hands-cross conditions, since in these two conditions, additional visual objects were also present near the coincidence point.

A further factor is the role of fixation or eye movements. Streaming perception is enhanced when observers track the visual stimulus either voluntarily or involuntarily (Kawabe & Miura, 2006; Watanabe & Shimojo, 1998). It is possible that specific hand postures might help the participants to fixate or focus better on the fixation point, but this line of reasoning cannot explain all the characteristic results obtained in the four experiments. In particular, for Experiments 1 and 2, there were similar visual information from the hands near the coincidence point, but nevertheless different results were obtained. In fact, we measured the eye movements in all the conditions of Experiment 1 by using a Tobii eye-tracker in three participants, but we did not find any systematic changes in the heat maps of fixations depending on the presence or absence of hands, or on the hand postures.

While the present study indicated that only the specific hand posture with the palms together could induce the HIB effect in some limited spatial range and suggested the importance of proprioceptive information from the own hands, other possibilities need to be examined. In particular, the effects of other hand postures, such as the fist-to-fist posture together with the sense of force, should be examined in future research.
